# Influence of the Deep Cryogenic Treatment on AISI 52100 and AISI D3 Steel’s Corrosion Resistance

**DOI:** 10.3390/ma14216357

**Published:** 2021-10-24

**Authors:** Patricia Jovičević-Klug, Tjaša Kranjec, Matic Jovičević-Klug, Tadeja Kosec, Bojan Podgornik

**Affiliations:** 1 Institute of Metals and Technology, Lepi pot 11, 1000 Ljubljana, Slovenia; tjasi.kranjec@gmail.com (T.K.); maticklug@gmail.com (M.J.-K.); bojan.podgornik@imt.si (B.P.); 2 Jožef Stefan International Postgraduate School, Jamova cesta 39, 1000 Ljubljana, Slovenia; 3 Max-Planck Institute for Iron Research, Max-Planck-Straße 1, 40237 Düsseldorf, Germany; 4 Laboratory for Metals, Corrosion and Anticorrosion Protection, Slovenian National Building and Civil Engineering Institute, Dimičeva ulica 12, 1000 Ljubljana, Slovenia; tadeja.kosec@zag.si

**Keywords:** steel, polarization, Raman spectroscopy, scanning electron microscopy, intergranular corrosion

## Abstract

The effect of deep cryogenic treatment (DCT) on corrosion resistance of steels AISI 52100 and AISI D3 is investigated and compared with conventional heat-treated counterparts. DCT’s influence on microstructural changes is subsequently correlated to the corrosion resistance. DCT is confirmed to reduce the formation of corrosion products on steels’ surface, retard the corrosion products development and propagation. DCT reduces surface cracking, which is considered to be related to modified residual stress state of the material. DCT’s influence on each steel results from the altered microstructure and alloying element concentration that depends on steel matrix and type. This study presents DCT as an effective method for corrosion resistance alteration of steels.

## 1. Introduction

Corrosion is a phenomenon plaguing numerous industries. Corrosion processes depend on exposure to selected media (seawater, rain, water, salt spray, etc.), industrial emissions and used material type [1,2]. Depending on the application of the final product, the corrosion resistance is usually tested to determine the acceptable rate of a corrosion attack on material before its exposure to actual corrosive media [3]. Although new advanced materials with superior corrosion resistance are developed every day, metals, particularly steels, are still the most common materials found in industrial applications. One of the possibilities to improve corrosion resistance of metals is by manipulating the metal surface by heat treatment [4] that tailors the material microstructure [5]. In the course of the past decade, a novel way of manipulating metallic surfaces has been developed by adding deep cryogenic treatment (DCT) as an accompanying heat treatment process [6]. During DCT, material is exposed to sub-zero temperatures (below −150 °C) for a certain period of time (usually between 24 and 48 h) in order to improve various material properties, including corrosion resistance [7,8]. A large portion of the change of macroscopic and physical properties is considered to be related to microstructural changes induced with DCT. However, studies of DCT phenomena and its effect on steel properties is predominantly focused on mechanical, fatigue and wear properties, while studies on corrosion resistance are very limited [9,10].

This study focuses on the evaluation of corrosion properties of selected bearing (AISI 52100) and tool steel (AISI D3) after application of DCT. AISI 52100 bearing steel is used in shafts and bearings, which can be exposed to corrosive environments such as water in the form of air moisture and condensation drops [11]. Consequentially, improved corrosion resistance and strength of the used material are favorable for improving the lifetime of the end-products. There is limited research of corrosion resistance of AISI 52100 in correlation with DCT. Study by Wang et al., 2014 [12], reported no significant change of the corrosion response of DCT samples of AISI 52100 in synthetic saltwater. In contrast, a number of studies [13,14], including DCT [15] were carried out on AISI D3 high-carbon, high-chromium tool steel, where high strength and superior resistance to wear and corrosion are required. However, the studies on the effect of DCT on the steel corrosion resistance provide only limited insight into the DCT mechanism responsible for the change in corrosion resistance [15,16]. The main explanation for changes in corrosion resistance after application of DCT on both steels is considered to be a consequence of an altered microstructure that is also determined with the change of hardness after DCT [8,17].

Due to the limited number of investigations related to the effect of DCT on corrosion resistance of steels and inconsistence of results, this study focuses on the investigation of two types of steels, AISI 52100 bearing steel and AISI D3 tool steel, in order to determine the effect of DCT on the relationship between mechanical, microstructural and corrosion properties. The selected steels were chosen based on the importance of corrosion resistance in bearing and tool industry, as well as due to their chemical composition (low and high chromium content). The aim is to determine DCT influence on steel’s corrosion resistance in seawater based on the alloying composition of the steel. Therefore, the main objectives of this study are: (i) to investigate the type and evolution of corrosion products in seawater for conventional (CHT) and deep cryogenic heat-treated AISI 52100 and AISI D3 steels and (ii) to determine the effect of DCT on the corrosion properties of the selected steels and to indicate differences originating from the steel type and composition.

## 2. Materials and Methods

### 2.1. Material and Heat Treatment

The steels used in the experiment are AISI 52100 (X) bearing steel and AISI D3 (Y) cold work tool steel (Table 1). Different samples were prepared from Ø20 mm rods for various testing techniques. Samples for monitoring the time evolution of corrosion were cut into 1 × 1 × 2.5 cm^3^ cuboids. Specimens for polarization measurements were prepared in the form of a disc with a diameter of 15 mm. Specimens were processed using two different heat treatments: the first was conventional heat treatment (CHT) with gas quenching and tempering, and the second was deep cryogenic treatment (DCT) (Table 2). DCT was performed right after quenching and before tempering. The soaking time of immersion in liquid nitrogen during DCT was 24 h at soaking temperature of −196 °C. The DCT warming/cooling rate was approximately 10 °C/min. In order to obtain high hardness or high fracture toughness, two subgroups of heat treatments were performed. In the first subgroup, the higher austenitization temperature and lower tempering temperature were selected in favor of higher hardness (X1–X2, Y1–Y2). In the second subgroup, lower austenitizing temperature and higher tempering temperature were selected favoring higher fracture toughness (X3–X4, Y3–Y4). The selection of heat treatment temperatures was based on the producer’s heat treatment processing recommendations for obtaining selected properties. Austenitizing, quenching and tempering were performed in a horizontal vacuum furnace IPSEN VTTC-324R with uniform high-pressure gas quenching using N_2_ gas at the pressure of 5 bars.

### 2.2. Methods

#### 2.2.1. Chemical, Metallographic and Reconstruction Analysis

Chemical composition of steels X and Y (Table 1) was determined by classical analysis of carbon and sulfur (ELTRA CS800) and X-ray fluorescence (XRF) with Thermo Scientific Niton XL3t GOLDD+ (Waltham, MA, USA). Size and corrosion measurement of samples were according to the ISO 11306:1998 standard (Corrosion of metals and alloys—Guidelines for exposing and evaluating metals and alloys in surface seawater). Samples were divided into two groups based on the two different heat treatments, CHT and DCT. Samples were mechanically grinded with silicon carbide (SiC) emery papers down to 1000 grit, polished with diamond pastes down to 1 µm particle size and then ultrasonically rinsed in ethanol. After the preparation, the samples were placed in a beaker with seawater. The chemical composition of the seawater (saltwater) used as medium is provided in our previous article by Jovičević-Klug et al., 2021 [19]. Samples were tested/immersed for 1 day and 7 days. Additional individual samples were also immersed for less than 1 day (1 h, 3 h and 7 h) to reveal the corrosion products temporal development. After the immersion, samples were dried and their surface was investigated by light optical microscopy (LM) (ZEISS Axio Vario, Carl Zeiss AG, Oberkochen, Germany) and scanning electron microscopy (SEM) (JEOL JSM-6500F, JEOL, Tokyo, Japan), to determine the corrosion products, their morphology, distribution and chemical composition (EDS-energy-dispersive X-ray spectroscopy). The measurements were then used to indicate the effect of different heat treatment (DCT/CHT) on the corrosion behavior based on the different seawater exposure. Due to the large number of results, only selected results are present in this study. In addition, the microstructure interpretation is based on previous study by Jovičević-Klug et al., 2021 [20]. The density and size of cracking of the steels’ surfaces after exposure to corrosive media was determined and analyzed by processing the SEM images with an area 1500 µm^2^ using ImageJ software. The size of cracks is determined by the average projected area of individual cracks, whereas the crack density is related to the number of cracks per area unit of material surface. The surface analysis of samples and evaluation was performed by focus variation microscopy with Alicona InfiniteFocus G4, Bruker Alicona, Graz, Austria. Statistical analysis of acquired data was performed in SPSS (PASWStatistics18, SPSS Inc., Chicago, Illinois, USA) and 3D data post processing with Origin 2021, OriginLab Corporation, Northampton, MA, USA. The samples for the statistical corrosion analysis of weight loss and corrosion rate were prepared according to C.3.1. G1-90 ASTM standard.

#### 2.2.2. Electrochemical Measurements

For the purpose of this research, the electrochemical measurements were performed in borate buffer with pH 10. The analysis was performed to indicate the key differences between the corrosion behavior of CHT and DCT steel samples in simulated alkaline environment induced by borate buffer that is often present as an ingredient of lubricants used in metal forming and load-bearing applications that typically require a pH value between 8.5–10 for metal residue removal [21,22]. The results were then used as boundary conditions to indicate the heat treatment groups with strongest and most interesting differences between CHT and DCT samples to further analyze their corrosive behavior in saltwater environment in order to indicate the relation of DCT and CHT on the steel corrosion behavior in naturally occurring alkaline environment. For the experiments, a three-electrode corrosion cell with a volume of 350 cm^3^ was used. Gamry, Ref 600+ equipment was used, operated with Gamry Framework Software. The electrochemical tests were performed as follows: 1 h stabilization at open circuit potential (OCP), following with linear polarization measurements at ±20 mV vs. OCP at a scan rate 0.1 mV/s. Polarization measurements were then performed starting from −0.25 V vs. OCP, and progressing in the anodic direction up to +1.2 V at a scan rate of 1 mV/s. All potentials are reported with respect to the Ag/AgCl scale. At least four measurements were performed for each heat treatment condition for both selected steels. Scatter diagrams of polarization resistance Rp were plotted in order to differentiate the groups of tested steels according to suggestions for electrochemical testing [23].

#### 2.2.3. Raman Spectroscopy

Following exposure, the surface morphology of samples was first inspected and analyzed with a scanning electron microscope JEOL JSM-IT500LV with Aztec Live Advanced ULTIM 65 detector (Oxford Instruments, Abingdon, UK) equipped with energy dispersive spectroscopy (EDX) at accelerating voltage of 20 kV, to select the most appropriate and representative regions for Raman spectroscopy. Raman spectra were obtained using a Horiba Jobin Yvon LabRAM HR800 Raman spectrometer (Longjumeau, France) coupled to an Olympus BXFM optical microscope (Tokyo, Japan). The measurements were performed using a 633 nm laser excitation line, a 100× objective lens and a 600 grooves/mm grating with a spectral resolution of 2 cm^−1^ per pixel. The power of the laser was set to 0.14 mW. A multi-channel air-cooled CCD detector was used, with integration times of between 20 and 30 s.

## 3. Results and Discussion

### 3.1. Electrochemical Measurements

**Figure 1:**
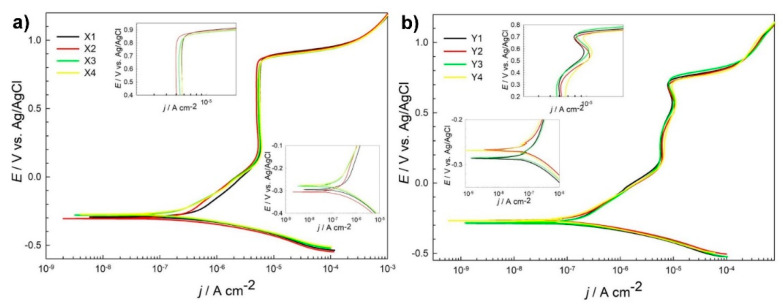
Potentiodynamic measurements for steel grade X (AISI 52100) (**a**) and steel grade Y (AISI D3) (**b**) in borate buffer pH 10 for conventional heat-treated samples (X1, X3, Y1 and Y3) and deep cryogenic heat-treated samples (X2, X4, Y2, Y4) at a scan rate 1 mV/s.

Potentiodynamic curves, as shown in Figure 1a, for different heat treatment subgroups of steel X (AISI 52100) are similar. However, certain differences were observed. *Ecorr* value varies from −0.276 for subgroup X4-DCT to the lowest value for subgroup X2-DCT. Corrosion current density *jcorr* is smallest for steel grade X3-CHT and is the highest for sample X1-CHT. Lower corrosion current densities correspond to lower value of breakdown potential *Eb* (Table 3). It is the highest, most positive (1.02 V) for steel Y3-CHT, which has the highest *jcorr* value (0.362 μA/cm^2^). The measurements indicate a potential difference in the corrosion properties for differently thermally treated samples. From Supplementary Material 1 one can differentiate among different groups of tested samples X. *Rp* value for DCT subgroup X2-DCT increases for 44% when compared to X1-CHT, while value of *Rp* for X4-DCT reduces for 48% after DCT when compared to X3-CHT.

Similar shape and electrochemical parameters were deduced from potentiodynamic curves for steel grade Y (Figure 1b), with the corrosion current densities (Table 3) being smaller when compared to steel samples grade X. However, *Rp* values in general are higher, but the scatter between the obtained results is higher as well (Supplementary Material 1). *Rp* value for DCT subgroup Y2-DCT reduces for 17% when compared to Y1-CHT, while value of *Rp* for Y4-DCT increases for 48% after DCT as compared to Y3-CHT (Table 3).

Polarization resistance (*Rp*) of AISI 52100 steel is improved by 44% with DCT treatment when using higher austenitization (870 °C) and lower tempering temperature (150 °C) (X1 -> X2; Table 2). Another observation of improved corrosion resistance induced by DCT is determined for steel AISI D3 austenitized at 950 °C and tempered at 300 °C (Y3 -> Y4; Table 2). *Rp* of sample Y4 was increased by 48% when compared to counterpart Y3. The reason behind the improved corrosion resistance in these subgroups is in the microstructure, which is discussed in detail in the paper of Jovičević-Klug et al., 2021 [20]. The microstructure reveals that for steel X, the carbide density increases for both heat treatments, but result in different ratio of the Fe_3_C (cementite) and Fe_7_C_3_ carbides. For X2 in comparison to X1, both cementite and Fe_7_C_3_ carbides increase, whereas for the X4, in comparison to X3, the amount of M_7_C_3_ carbides increases at the expense of reduced amount of cementite. This can well explain the trend of *Rp* values, as the increased number of precipitates increases the corrosion resistance of the X2 sample due to higher nobility of the surface caused by higher number of carbides. For X4 the corrosion resistance decreases despite the increased carbide formation, as the M_7_C_3_ are less stable compared to cementite [24,25,26]. For steel Y, the microstructural changes after DCT do not only impact the carbide density and their ratio, but also change the content of retained austenite. For sample Y2, in comparison to Y1, the content of retained austenite drops from 15 vol.% to 2 vol.% and the carbide density increases (cumulatively by 4 vol.%). The increased carbide density should increase the corrosion resistance of the steel as seen for steel X. However, the significant drop in austenite dominates the decrease of corrosion resistance of sample Y2, since austenite promotes the corrosion resistance in comparison to martensite [27]. For Y4, in comparison to Y3, both samples have a small amount of retained austenite present as well as similar volumetric amounts of carbides, but the ratio of carbides is again significantly different as observed for steel X with the lower austenitizing and higher tempering temperature heat treatment. For sample Y4, the amount of M_23_C_6_ carbides increases by nearly 40%, whereas the amount of M_7_C_3_ carbides decreases by 26% (corresponding to 6 and 4.5 vol.%, respectively). This significant shift in carbide ratio is considered to be the reason for the increased corrosion resistance, since the M_23_C_6_ carbides are considerably more stable due to their face centered cubic structure in comparison to the M_7_C_3_ carbides, which have an orthorhombic crystal structure. Furthermore, the carbide number is significantly higher, as the average size of M_23_C_6_ carbides is much smaller compared to the M_7_C_3_ carbides (see [20]), which also relates to the more homogeneous distribution of the carbides and shorter carbide to carbide distance. In turn, this and the effect of more homogeneous distribution of alloying elements after DCT [28] also reduces the formation of localized depletion zones of alloying elements, such as Cr that can cause local increased corrosion propagation [27]. All of these features are considered to improve the corrosion resistance of steel Y4 and results in a considerable increase of *Rp* by nearly 50% compared to Y3.

Based on the potentiodynamic measurements that disclose smaller changes in the Rp for groups X1, X2 and Y1, Y2, and due to their different hardness and fracture toughness changes with DCT, these samples were selected for further investigation. These samples were also chosen in order to see the differing effect of DCT induced microstructural changes (carbides and retained austenite) on the overall corrosion response of the treated steels. The further analysis of these samples with regard to the DCT induced changes of corrosion products development and corrosion propagation will provide insight into the effect of DCT on the corrosion response of the selected steels with regard to the modified microstructure and mechanical properties.

### 3.2. Characterization of Corrosion Products with SEM

**Figure 2:**
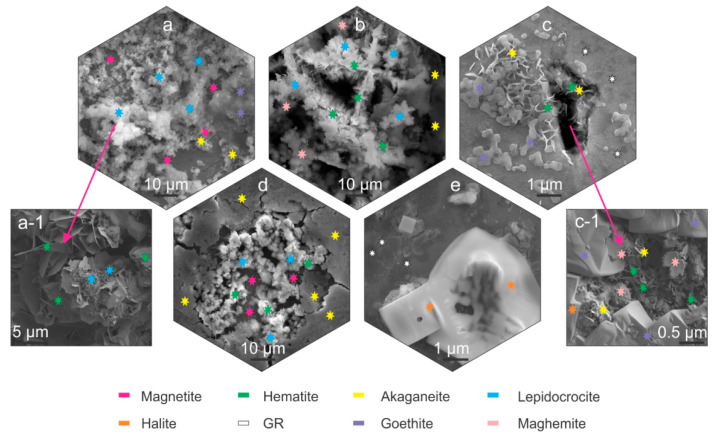
Scanning electron microscope-SEM micrographs of corrosion products marked with stars indicating the different corrosion products. Magnetite, akaganeite and lepidocrocite (**a**). Magnified image of hematite and lepidocrocite (**a-1**). Maghemite, akaganeite, hematite and lepidocrocite (**b**). Akaganeite, goethite; green rust (GR) and hematite (**c**). Magnified image of maghemite, akaganeite, goethite, hematite and halite (**c-1**). Akaganeite, magnetite, lepidocrocite and hematite (**d**). Green rust and halite (**e**).

**Figure 3:**
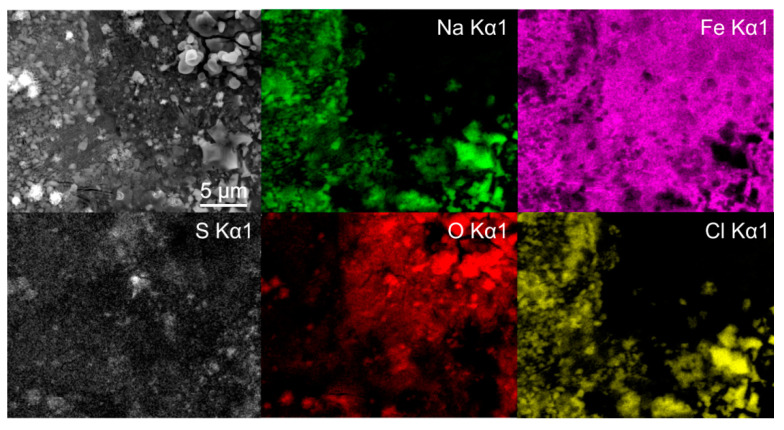
Energy dispersive X-ray spectroscopy mapping of deep cryogenic heat-treated (DCT) steel Y after 1 day immersion time.

Microscopic analysis with SEM was performed in order to identify possible corrosion products and to further provide high-resolution images of corrosion products and their temporal evolution. The different corrosion products are correlated through their coloration, identified with LM, morphology determined with SEM and chemical composition with EDS. The corrosion products were further analyzed with Raman spectroscopy. The observations allowed the understanding of the corrosion products development of individual samples (Figure 2 and Figure 3, Supplementary Material 4, Supplementary Material 5).

The SEM investigation determined that the corrosion products for all samples formed predominantly in segments across the samples surface, as observed also with LM analysis (Supplementary Material 2, Supplementary Material 3). For bearing steel X, lepidocrocite forms preferentially across the steel surface (Figure 2a,b), regardless of the heat treatment. The lepidocrocite occasionally is covered with hematite platelets and needles (Figure 2(a-1) and Figure 2b, respectively) that form, due to dehydration of lepidocrocite [29]. Underneath lepidocrocite, akaganeite and maghemite are present, which form an intertwined corrosion layer (Figure 2b). Goethite was found to form intermediately in patches surrounding the regions covered with lepidocrocite (Figure 2a). For tool steel Y, the corrosion products are scarcely formed. The prevailing element of the corrosion products is green rust that thinly covers the surface. Occasional formation of small patches of akaganeite and goethite form around local excessive corrosion damage, indicated as pits, and cracks induced by local residual stress (Figure 2c). These surfaces are also randomly seeded by needle formed hematite, as seen in Figure 2c. The inner parts of cracks are covered with hematite platelets and maghemite florets, whereas the outer regions of the cracks are mainly covered by goethite, accompanied by the occasional presence of akaganeite (Figure 2(c-1)).

On the other hand, pits display mainly akaganeite around the pits, whereas the center of the pit is covered with lepidocrocite with occasional hematite situated within the lepidocrocite structures (Figure 2d). The underlying parts of the corrosion product layer display mainly magnetite which forms, due to anoxic conditions in the deeper parts of the pits. In the areas, where the corrosion products are less pronounced, the green rust displays a smoother surface and resembles more plate-like formations (Figure 2d). Such green rust is considered to be GR I type, which forms when the OH- groups are replaced by chlorine ions [19] and is a different type to the sulphate variant determined in other areas with Raman spectroscopy, which is presented in the next section. These regions also exhibit growth of considerably large halite structures (Figure 2c,d), which supports the claim of formed GR I, as the halite crystals are indicators of increased concentration of Cl^-^ ions.

To further identify the formation of the different types of green rust, EDS mapping was performed after 1 day immersion. The maps confirm the presence of two different types of green rust, which can be seen in Figure 3. The sulfur map shows a clear concentration increase on the sites with more textured surface (darker regions in SEM image), which result from the GR II type. The regions with reduced sulfur concentration correspond morphologically to the GR I type. The Fe and O maps indicate no significant difference in the oxidation of the iron matrix confirming that the regions are covered by green rust. The chlorine signal from these regions cannot be clearly seen from the map, due to the high signal of the surrounding halite structures. Nevertheless, local EDS analysis confirms the increased chlorine concentration in the darker regions, with an average 5 atm.% of Cl and only 1 atm.% Na, confirming that halite is not the only source of chlorine (other salt-forming elements such as K, Mg and Ca were not detected in the regions). In the regions with increased sulfur concentrations, chlorine is detected in trace amounts that correlate to trace amount of halite. The analysis confirms that there is no specific relation between the different microstructural elements and the green rust formation. The large halite structures also give a clear indication that the salt ion exchange also has no favored positions in terms of microstructural elements.

When comparing steel X and Y, a lesser amount of corrosion products develops on steel Y, as expected due to differences in chemical composition and much high amount of Cr in steel Y. The differentiation between the CHT and DCT state was further studied with SEM investigation for samples exposed to seawater for 1 h, 3 h and 7 h. The DCT samples of steel X (Supplementary Material 4a–f) generally display more akaganeite across the steel surfaces within the first 7 h of immersion compared to their CHT counterparts (Supplementary Material 4g–l). The steel X DCT samples also display goethite patches that overgrow the akaganeite with increasing immersion time. Regions with slower corrosion development clearly show the formation of green rust (Supplementary Material 4d), which are overgrown with magnetite patches. With further immersion time, the DCT samples develop localized regions of increased corrosion that result in the formation of pits topped with hematite and lepidocrocite (Supplementary Material 4c–f). In contrast, the CHT samples of steel X develop lepidocrocite faster and on larger regions (Supplementary Material 4g–i). The lepidocrocite formations hold hematite needles, which are mostly situated underneath lepidocrocite structures, as seen in Supplementary Material 4h–i. Nevertheless, the CHT samples of steel X also display regions with slower corrosion development that display green rust covered by goethite and magnetite (Supplementary Material 4j,k). Furthermore, the CHT samples also display pits covered by lepidocrocite and hematite. However, the edges are covered with thicker akaganeite, which is indicated by the released corrosion scales from the pits (Supplementary Material 4l). In general, for steel X the DCT samples exhibited slower corrosion development and formation of lepidocrocite is less abundant. The propagation of excessive local corrosion damage is considered to be an influencing factor that causes the difference between DCT and CHT samples and will be further analyzed and discussed in the next section. For tool steel Y, no significant difference is revealed in the corrosion product evolution, which is considered to be related to the slower corrosion development of steel Y (Supplementary Material 5a–f). Both DCT and CHT samples display a predominant presence of green rust that eventually becomes covered by other corrosion products such as magnetite, goethite, akaganeite and lepidocrocite. The only difference is observed in relation to the occurrence of corrosion product groupings, which more densely occur for the CHT samples. The possible explanation is once more related to the excessive local corrosion damage propagation, which can be influenced by DCT through the homogenization of the microstructure [30].

**Figure 4:**
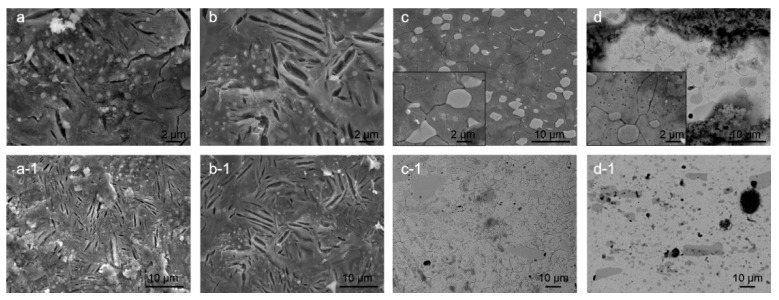
Scanning electron microscopy images of conventional heat-treated (**a**) and deep cryogenic heat-treated (**b**) steel X and conventional heat-treated (**c**) and deep cryogenic heat-treated (**d**) steel Y. The images display dependency of the surface cracking, considered to be related to residual stresses, to the microstructure and heat-treated state. The immersion time for steel X samples was 3 h, whereas for steel Y, the immersion time was 1 day.

Another aspect of the corrosion behavior is also surface cracking. The cracking was observed after gentle rinsing of the corroded surface with deionized water and removal of corrosion products. The observed cracking is considered to be related to the residual stresses within the microstructure of heat-treated samples. For bearing steel samples X1 and X2, the cracking occurs mostly between the ferrite/pearlite/cementite structures, which results from the preferential corrosion attack of the ferrite (α-iron) regions situated between cementite regions (Figure 4a,b). The major difference between the CHT and DCT treated samples is the size and density of the cracks. The cracks form with lower density (approx. 2× less) within the DCT samples, but the cracks are on average 2× wider compared to the cracks formed in CHT counterpart. The cracks usually open along the length of the pearlitic lamella and do not propagate into the next ferritic region (differently oriented lamella). For DCT sample no specific network of cracks was observed, whereas for CHT sample additional local networks of cracks are sometimes formed (Figure 4a). The cracks do not seem to bridge individual carbide precipitates, but do follow a certain form that relates to the boundaries between the different ferrite and perlite regions. Due to this, it is assumed that the lack of crack networks for DCT sample has positive effect on the corrosion resistance by reducing the chance of corrosion damage propagation and delamination. The different size and density of cracks should also give a positive contribution to the corrosion behavior of DCT samples, as the cumulative newly opened surface is smaller for the DCT sample as compared to the CHT counterpart. It is postulated that the reduced crack density for DCT sample is a result of the more homogeneous microstructure, which reduces the density of defects and weaker point’s density. This also goes hand in hand with the fracture toughness, as the reduced number of cracks should correlate to the increased fracture toughness of the DCT sample (sample X2) compared to the CHT sample (sample X1) (Table 2).

In contrast, the surface cracking, proposed to be a result of residual stress state, for both heat-treated variants of tool steel Y is determined to form in a similar pattern that follows the primary austenite grain boundaries and the Cr carbide boundaries (see Figure 4c,d). The comparison of Y1 and Y2 samples reveals that the DCT sample (Figure 4d) exhibits thinner cracks, which are less interconnected than the cracks found in CHT sample (Figure 4c). This feature is considered to positively affect the corrosion behavior of DCT samples, as the thinner cracks and reduced crack interconnection leads to reduced amount of large open voids that could potentially lead to local excessive corrosion damage and corrosion propagation. This form of cracking has no relation to the reduced fracture toughness for the DCT sample (Table 2), as the lower fracture toughness should result in the formation of larger cracks and/or density of cracks. The possible explanation is that DCT not only changes the microstructure [20], but also changes the residual stress of the material. It has been proven that for martensite steels, reduced tensile residual stress or even a change in the character of the residual stress state from tensile to compressive stresses may occur after DCT [31,32]. Furthermore, in previous publication, a similar effect has been confirmed on another steel type in connection to the corrosion behavior and stress state of the material [19]. Currently the research of residual stress modification with DCT for these steels is still underway and will be discussed and analyzed in an upcoming publication.

**Figure 5:**
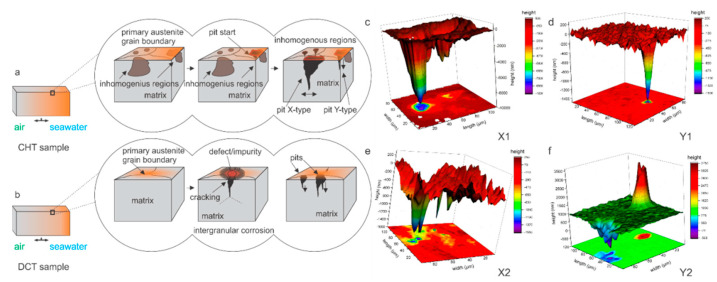
(**a**,**b**) Graphical representation of pit growth regarding heat-treated state of investigated steels conventional (CHT) and deep cryogenic (DCT) heat treatment. CHT sample pit growth resulting from direct vertical grain attack within inhomogeneous regions (**a**). DCT sample pit growth resulting from intergranular corrosion on defect/impurity portions of the primary austenite grain boundaries (**b**). (**c**,**d**) Three-dimensional measurements of pits in CHT X1 and Y1 samples. (**e**,**f**) Three-dimensional measurements of pits in DCT X2 and Y2 samples.

The comparison of CHT and DCT samples microstructure confirms that the homogeneity of the matrix through the distribution of alloying elements [17,30] and the reduction of localized defects with DCT is the main mechanism that reduces the localized excessive corrosion damage and corrosion development [10]. This modification also relates to the different initialization of the pits and their growth mechanism, as portrayed in Figure 5. The different formation mechanisms also directly affect the depth of the corrosion damage and the expansion of the damage. In the case of CHT, the limitation is related to the matrix (limit imposed by carbides) and by the inhomogeneous distribution of alloying elements, whereas for DCT samples, the limitation is the verticality of the attacked grain boundary. This also directly influences the width of the pit, as for the direct grain attack, the matrix can be additionally eroded from the sides of the pit, resulting in increased pit width (pit X-type in Figure 5a,c). However, if the pit only expands in the exposed upper part and is continuously reduced down with the depth of the pit section (pit Y-type in Figure 5a,d). It is proposed that in the case of intergranular attack for DCT samples (Figure 5b,e,f), the crack progresses extensively slower, when the grain boundary diverts from the orthogonal orientation to the sample surface. This occurs, since the corrosion attack is limited to the grain boundary and to the exposure of the crack opening to influx of oxidative media, which becomes limited with the change of crack propagation orientation with respect to the sample surface.

### 3.3. Characterization of Corrosion Products with Raman Spectroscopy

**Figure 6:**
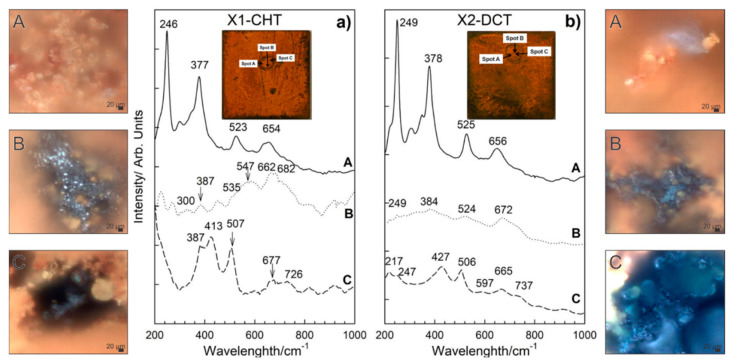
Raman spectra of corrosion products formed on steel samples X1-CHT (**a**) and X2-DCT (**b**) after 1 day exposure to seawater. Both spectra are also accompanied by light microscopy images (**A**–**C**) of corrosion products obtained at 1000× magnification.

Different morphologies of corrosion products were identified and confirmed based on SEM analysis for both steels. Raman spectra of sample bearing steel X1-CHT indicated fine structured orange corrosion products, represented by spot A in Figure 6a, revealed the most intensive bands at 140, 246, 377, 523 and 654 cm^−1^, indicating the presence of orthorhombic lepidocrocite, γ-FeOOH [33]. Regions of more compact agglomerates of greyish corrosion products as observed by optical microscope (Spot B), display complex Raman spectra, which are correlated to the presence of magnetite Fe_3_O_4_, goethite α-FeOOH and maghemite γ-Fe_2_O_3_. Broad bands in Raman spectra exhibited by magnetite were found at 300, 403, 535, and 662 cm^−1^. However, the 403 cm^−1^ band was not well expressed, which is probably a result of the overlap with goethite band at 387 cm^−1^. The most intensive bands of goethite were observed at 242, 387 and 682 cm^−1^. Maghemite (γ-Fe_2_O_3_) was revealed from the most intensive bands at 346, 500 and 718 cm^−1^. Orthorhombic goethite α-FeOOH and monoclinic akaganeite β-FeOOH were found in the lower sections of the corrosion products layer (spot C, Figure 6a), closely attached to the steel surface. Goethite was revealed from most intensive bands occurring at 387, 507, 677 cm^−1^, while akaganeite β-FeOOH can be recognized from broad bands at 299, 387, 413, 538 and 726 cm^−1^ [33].

On the deep cryogenic heat-treated bearing steel X (X2-DCT), three distinctive morphologies were found and analyzed (Figure 6b). Raman spectra on spot A (Figure 6b), where fine sized corrosion products were found, revealed the presence of lepidocrocite. Raman spectrum was well defined and the bands of lepidocrocite are well expressed. Lepidocrocite has been found to be unstable and porous, and tends to dissolve and transform to other oxide and hydroxide forms over time, thus offering weaker corrosion protection. On spot B, a compact dark corrosion product goethite, α-FeOOH, was found with the main bands at 250, 383, 527 and 672 cm^−1^. From the Raman spectrum on spot C (Figure 6b), the typical corrosion products grown in alkaline saline environment could not be revealed. However, the band at 217 cm^−1^ and the two strongest bands at 427 and 506 cm^−1^ point to the presence of green rust (GR), a product that is found on steel surfaces in seawater [34]. Green rust is an iron (II) and iron (III) double hydroxide corrosion product of steel in seawater, where OH- ions can be substituted with SO_4_^2−^, NO_3_^−^ or Cl^−^ ions [25], moreover, sometimes Mg^2+^ is incorporated instead of Fe^2+^ [35,36]. The measured spectra is determined to be related to green rust type GR II, which is the hydroxysulphate form GR(SO_4_^2−^), based on the comparison with a similar spectra reported by Lanneluc et al., 2015 [35].

**Figure 7:**
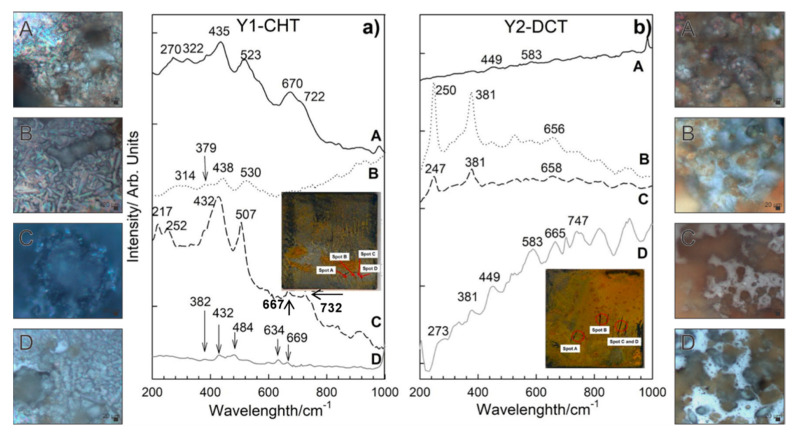
Raman spectra of corrosion products formed on steel samples Y1-CHT (**a**) and Y2-DCT (**b**) after 1 day exposure to seawater. Both spectra are also accompanied by light microscopy images (**A**–**D**) of corrosion products obtained at 1000× magnification.

Corrosion products formed on Y1-CHT steel after 1 day immersion to seawater (Figure 7a) are different from X1-CHT steel. They are less abundant. Raman spectra on orange areas indicate towards the presence of akageneite, β-FeOOH monoclinic, with bands at 386, 413, 538 cm^−1^ and has a broad and most intensive band at 726 cm^−1^. Relatively intensive bands at 434 and 518 cm^−1^ are attributed to sulphated green rust, with a shift of 20 cm^−1^ [35]. On spot B (Figure 6a), lepidocrocite and akaganeite were found, again Raman bands at 442 and 524 cm^−1^ could be related to the GR(SO_4_^2−^) [35]. Dark areas of corrosion products on spot C revealed the presence of hematite and maghemite. Bands at 427 and 507 cm^−1^ are visible as well, which point to GR II. A thin layer with no visible agglomerates of corrosion products at the grey area on spot D revealed the presence of hematite α-Fe_2_O_3_ and orthorhombic goethite, α-FeOOH. On spot A of sample Y2-DCT (Figure 7b), magnetite was found with bands at 321, 513 and 670 cm^−1^. On spot B and C, lepidocrocite γ-FeOOH was determined, with the most intensive bands at 247, 381, 526 and 662 cm^−1^. Raman spectra on spot D is very complex, apart from lepidocrocite and magnetite, the rest of the corrosion products is difficult to define, due to the overlap of the bands. It is suggested that a mixture of other hydroxides, such as akageneite and goethite, could be present.

The Raman spectroscopy analysis confirmed the presence of various corrosion products, which are differently distributed for steel X and Y. With regard to the CHT and DCT state of the steels, it is clear that the corrosion products form differently. Steel X displayed larger presence of darker-blue regions, which correspond to green rust GR II and goethite. The darker regions were also found in CHT variant, but more scarcely, without indicated green rust and accompanied by akageneite. Furthermore, the presence of lepidocrocite was determined to be higher for CHT sample compared to its DCT counterpart. This illustrates that the corrosion advances faster for CHT sample of bearing steel X, as green rust is usually a precursor for the formation of other corrosion products such as goethite and akageneite, over which lepidocrocite grows [1]. For tool steel Y the clear difference between the product formations is not visible in the sense of the formation of different corrosion products. However, the distribution of the individual regions reveals that the CHT sample exhibits a general coverage of the surface with either green rust or hematite with little non-covered surface. On the other hand, the DCT sample exhibits many patches of the steel surface, which are not covered by any corrosion products (bright parts in Figure 7b). This suggests that for tool steel, the corrosion is retarded with DCT to such a degree that segments of the surface have not even begun to corrode in contrast to the surface of the CHT sample. With these results, Raman spectroscopy also confirms the general improvement of the corrosion resistance with DCT for both steels, bearing and tool steel.

### 3.4. Statistical Corrosion Analysis

**Figure 8:**
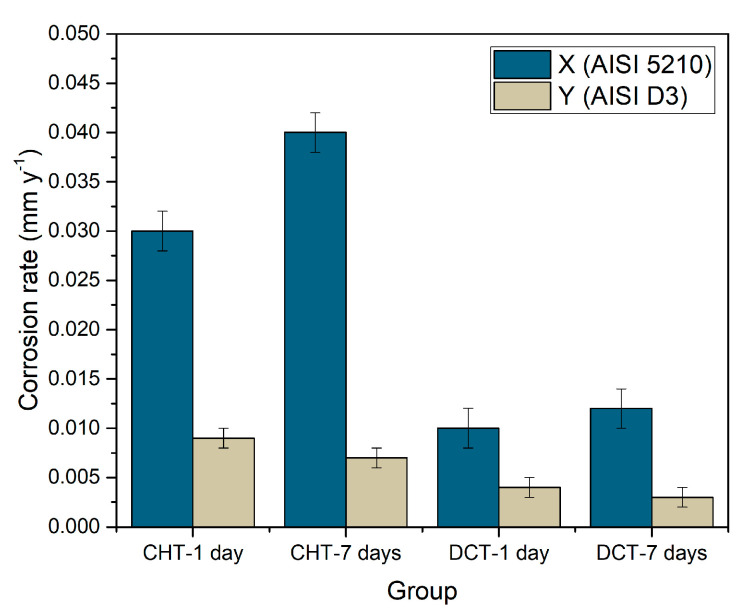
Graphical representation of corrosion rate of conventional and deep cryogenic heat-treated samples of steel X (X1–X2) and Y (Y1–Y2).

In order to determine the corrosion rate, weight loss during the immersion was measured for samples X1–X2 and Y1–Y2. The weight of each sample (10 samples per each heat treatment—CHT/DCT) was measured before and after exposure in seawater for 1 day and 7 days. Corrosion rate (Figure 8) was calculated in order to determine the influence of the different heat-treated state, CHT and DCT, on final corrosion properties (corrosion resistance). Corrosion rate was calculated according to the Equation (1):

(1) $$Corrosion\;rate\;\left(\frac{mm}{y}\right)=87.6\times \frac{W}{DAT}$$

where 87.6 is constant for steel, *W* is weight loss (mg), *D* is density of the steel (g/cm^3^), *A* is exposed area (cm^2^) and *T* is time (h). The corrosion rate of CHT and DCT samples can be seen in Figure 8.

The average corrosion rate for bearing steel X after 1 day is 0.03 ± 0.002 mm/y and for 7 days immersion is 0.04 ± 0.002 mm/y if conventionally heat-treated (CHT) and 0.01 ± 0.002 mm/y (1 day) and 0.012 ± 0.002 mm/y (7 days) for DCT treated, representing 56% lower corrosion rate. Similar observation is made for tool steel Y (AISI D3). The corrosion rate for CHT samples is 0.008 ± 0.001 mm/y and 0.003 ± 0.000 mm/y for DCT samples. The average corrosion rate of DCT samples is 65% lower than for CHT samples, confirming higher corrosion resistance of DCT treated samples. The data indicates that there is a reverse trend of corrosion rate with time, when comparing both steels, regardless of the application of DCT. For bearing steel X the corrosion rate increases with time, whereas for steel Y, the corrosion rate decreases. This is an interesting phenomenon, which can be correlated with the matrix phase and Cr content. Steel X has a perlite matrix, which deteriorates faster with increasing immersion time, which correlates well with the microscopic observations that indicate progressive corrosion attack through matrix cracking (separation of cementite and ferrite) and localized excessive corrosion damage. On the other hand, steel Y has a martensitic matrix, which has a high Cr content. It is believed that with immersion time, the corrosion progression is slower as the attacked regions develop passivation layering that prohibits further corrosion attack and degradation of the steel surface. Similar observation of reduced corrosion rate with immersion time in corrosive environment has been observed for similar steel [36].

## 4. Conclusions

In this study, various techniques were used in order to determine the effect of DCT on corrosion resistance of two steel types, AISI 52100 bearing steel and AISI D3 cold work tool steel.

(1) The corrosion resistance determined through *Rp* measurements indicates a varying effect of DCT on the corrosion response of selected steels based on the heat treatment parameters. The varying contribution was associated to the different DCT induced microstructural changes in relation to carbide precipitation, ratio of different carbide types and quantity of retained austenite.
(2) Corrosion products observed in samples regardless of the heat treatment history are goethite (α-FeOOH), akaganeite (β-FeOOH), lepidocrocite (γ-FeOOH), magnetite (Fe_3_O_4_), hematite (α-Fe_2_O_3_), maghemite (γ-Fe_2_O_3_) and green rust (GR I and GR II). The major difference is observed in relation to the occurrence of corrosion product groupings, which form more densely for the CHT samples. The phenomenon is attributed to the local excessive corrosion damage, which can be influenced by DCT through the homogenization of the microstructure.
(3) The comparison between DCT and CHT samples revealed that DCT samples exhibited lower number of cracks (for steel X) and thinner surface cracks (for steel Y) suggested to be induced by residual stress. Furthermore, the caracks are less interconnected than the cracks found in CHT samples. This feature is considered to positively affect the corrosion behavior of DCT samples, as the thinner cracks/lower number of cracks and reduced crack interconnection lead to a reduced amount of large open voids that contribute to increased corrosion damage and corrosion propagation.
(4) The study provides clear evidence on the corrosion improvement (55–65%) of two different steel grades with the exposure to DCT in seawater, with the efficiency depending on the steel type and heat treatment strategy (austenitizing and tempering temperature). As such, DCT represents an effective method to increase the corrosion resistance of steels throughout the material’s volume in a simple yet effective heating procedure, which has the potential to be applied on an industrial scale.

## Figures and Tables

**Table 1:** Chemical composition of AISI 52100 and AISI D3 steels (wt.%).

Steel Grade	C	Mn	S	Cr	Mo	V	Si	Ni	Fe
AISI 52100 (M.N. 1.3505/EN 100Cr6)	0.93	0.41	0.004	1.40	-	-	0.30	-	base
AISI D3 (M.N. 1.2080/EN X210Cr12)	2.07	0.35	0.002	12.40	0.13	0.16	0.23	0.11	base

**Table 2:** Heat treatment of AISI 52100 (designated as X) and AISI D3 steel (designated as Y) samples with corresponding hardness and fracture toughness values, where CHT stands for conventionally heat-treated and DCT for deep cryogenic heat-treated samples. The hardness and fracture toughness were obtained by standardized procedures, explained in our previous publication [18].

Steel	Subgroup	Austenitization (°C/min)	DCT (°C/h)	Tempering (°C/h)	Hardness (HRC)	Fracture Toughness (MPa√ m)
AISI 52100 (X)	X1-CHT	870/30	-	150/1	39 ± 0.3	24 ± 1.8
	X2-DCT	870/30	−196/24	150/1	38 ± 0.3	27 ± 1.6
	X3-CHT	830/30	-	350/1	31 ± 0.5	19 ± 2.5
	X4-DCT	830/30	−196/24	350/1	30 ± 0.5	15 ± 1.9
AISI D3 (Y)	Y1-CHT	980/20	-	350/2	64 ± 0.4	39 ± 0.4
	Y2-DCT	980/20	−196/24	350/2	66 ± 0.3	40 ± 0.4
	Y3-CHT	950/20	-	300/2	58 ± 0.5	17 ± 0.7
	Y4-DCT	950/20	−196/24	300/2	59 ± 0.5	18 ± 0.5

**Table 3:** Electrochemical parameters, deduced from potentiodynamic curves and linear polarization measurements.

Sample	Ecorr (V)	jcorr (μA/cm^2^)	Eb (V)	Rp (kΩ cm^2^) *
X1	−0.295	0.362	1.02	32.3
X2	−0.306	0.250	0.837	46.6
X3	−0.280	0.199	0.818	89.4
X4	−0.276	0.211	0.835	46.1
Y1	−0.286	0.149	0.693	109
Y2	−0.267	0.229	0.673	90.3
Y3	−0.284	0.153	0.711	92.6
Y4	−0.268	0.121	0.697	177

* Value, deduced from linear polarization measurements, scan rate 0.1 mV/s, Supplementary Material 1.

## Data Availability

The raw/processed data required to reproduce these findings cannot be shared at this time as the data also forms part of an ongoing study.

## Supplementary Materials

The following are available online at https://www.mdpi.com/article/10.3390/ma14216357/s1, Supplementary Material 1, Supplementary Material 2, Supplementary Material 3, Supplementary Material 4 and Supplementary Material 5.
