# Supplementary figures and images for: Influence of the Deep Cryogenic Treatment on AISI 52100 and AISI D3 Steel’s Corrosion Resistance
Source: Materials (Basel). 2021 Oct 24;14(21):6357. doi: 10.3390/ma14216357 (PMC8585173; doi:10.3390/ma14216357)

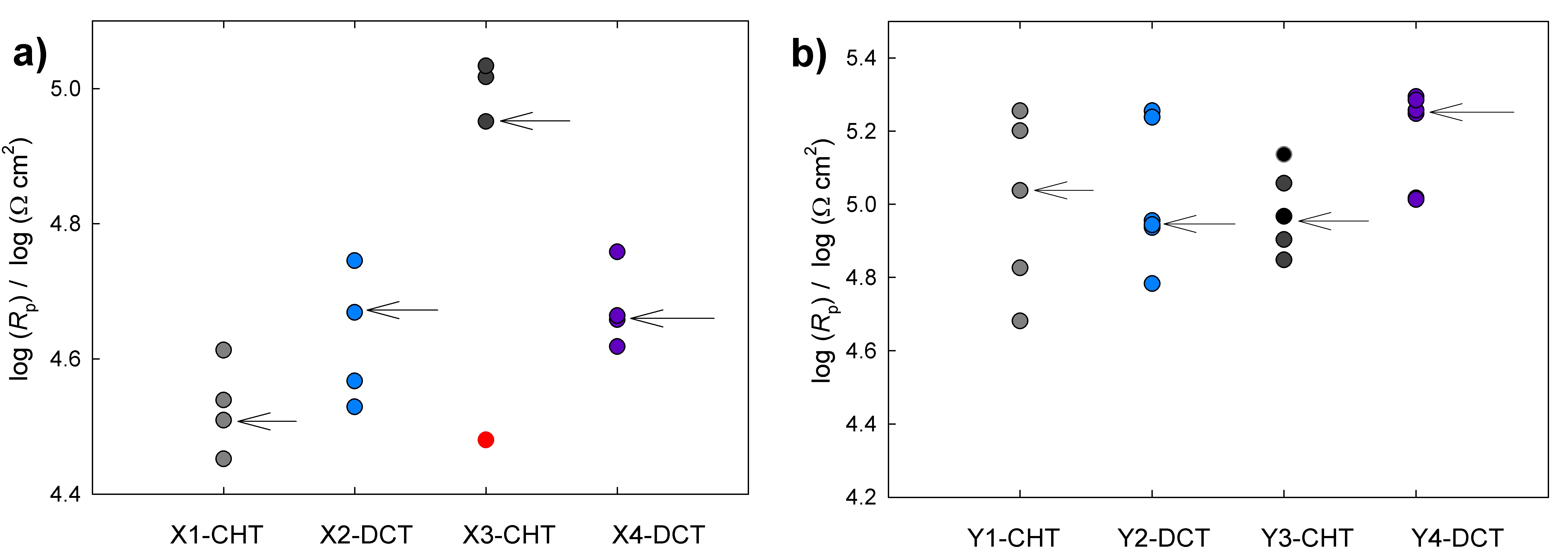

Supplement: Supplementary file 1 [file materials-14-06357-s001.zip › SM/SM1.jpg]

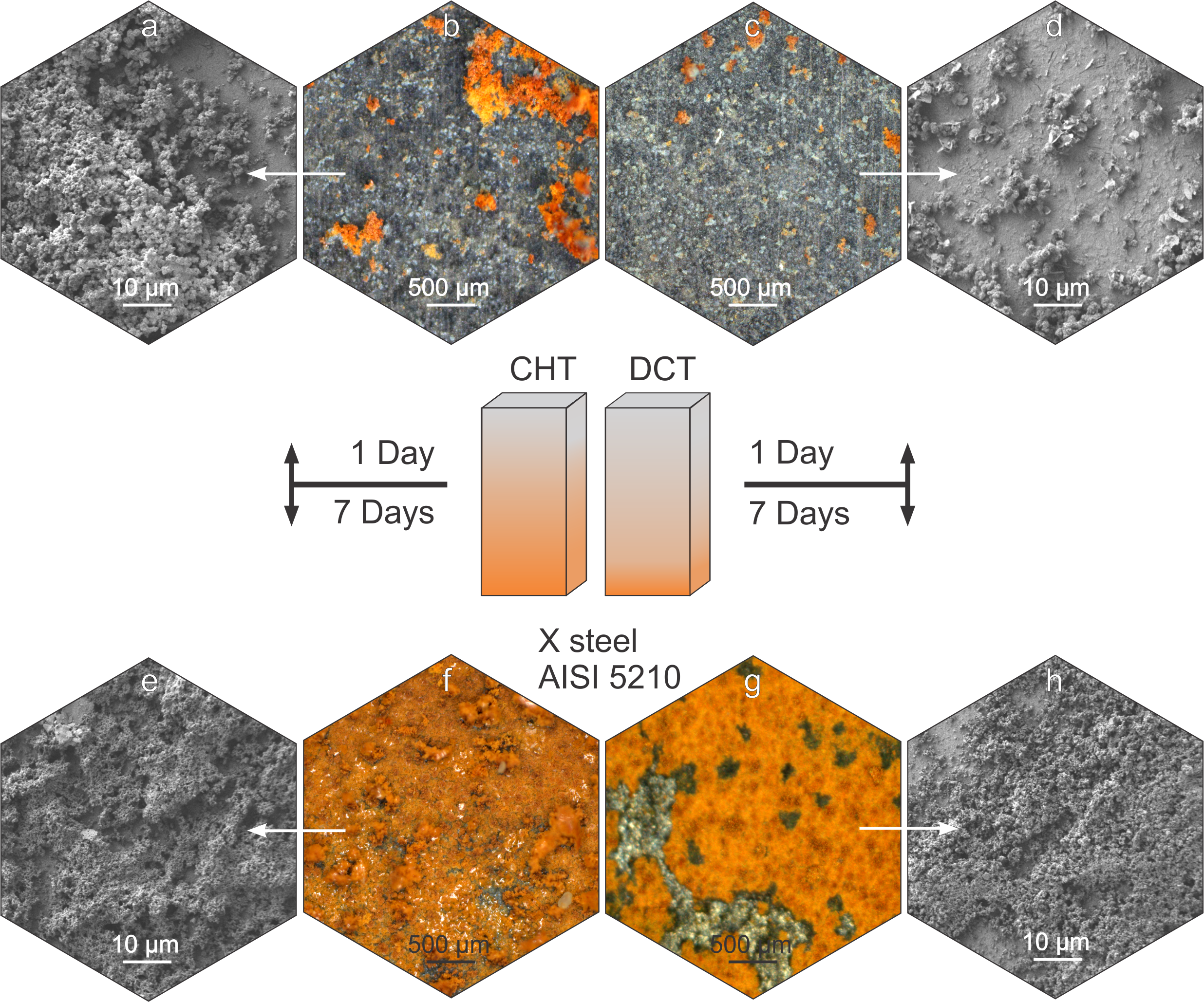

Supplement: Supplementary file 1 [file materials-14-06357-s001.zip › SM/SM2.png]

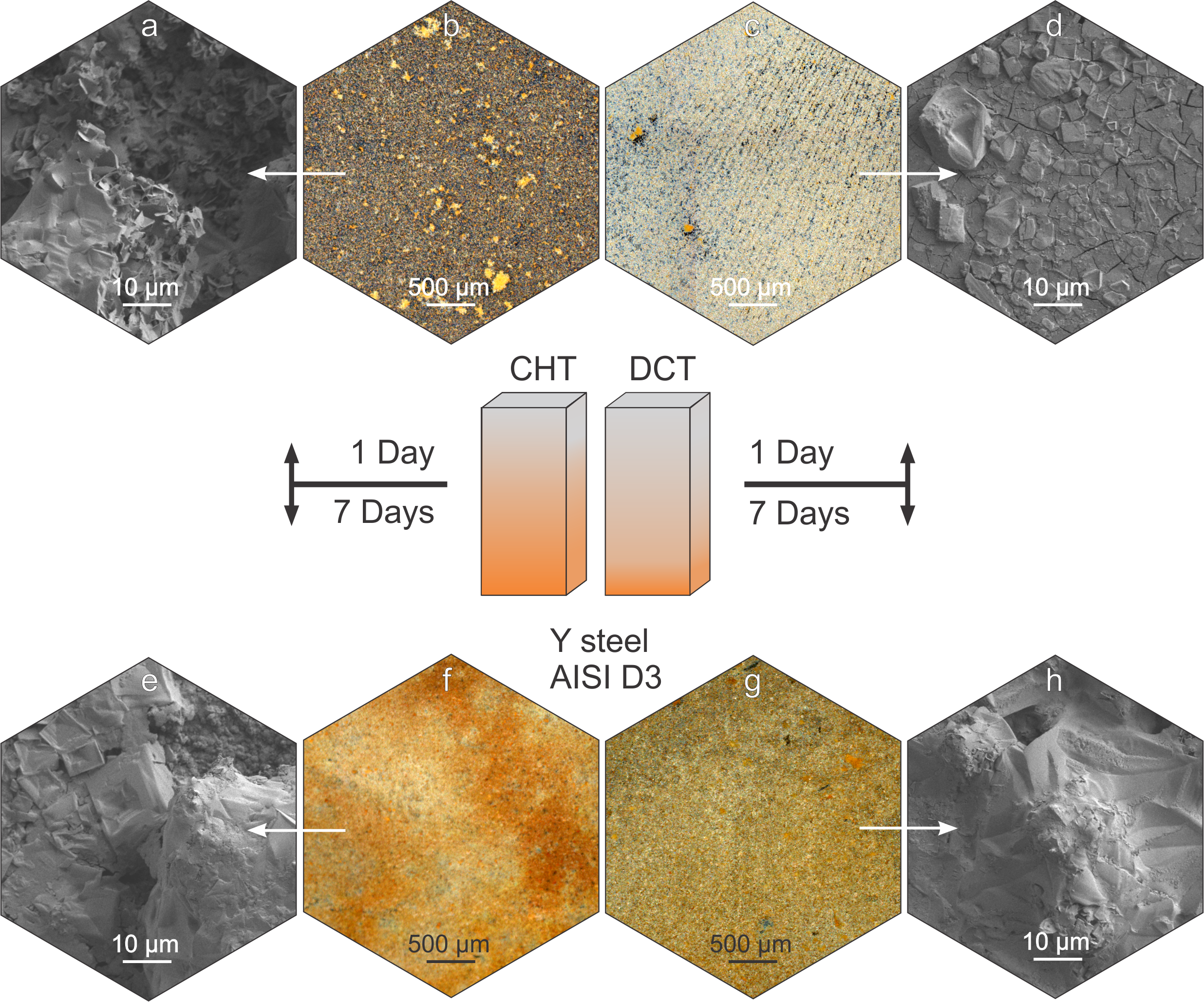

Supplement: Supplementary file 1 [file materials-14-06357-s001.zip › SM/SM3.png]

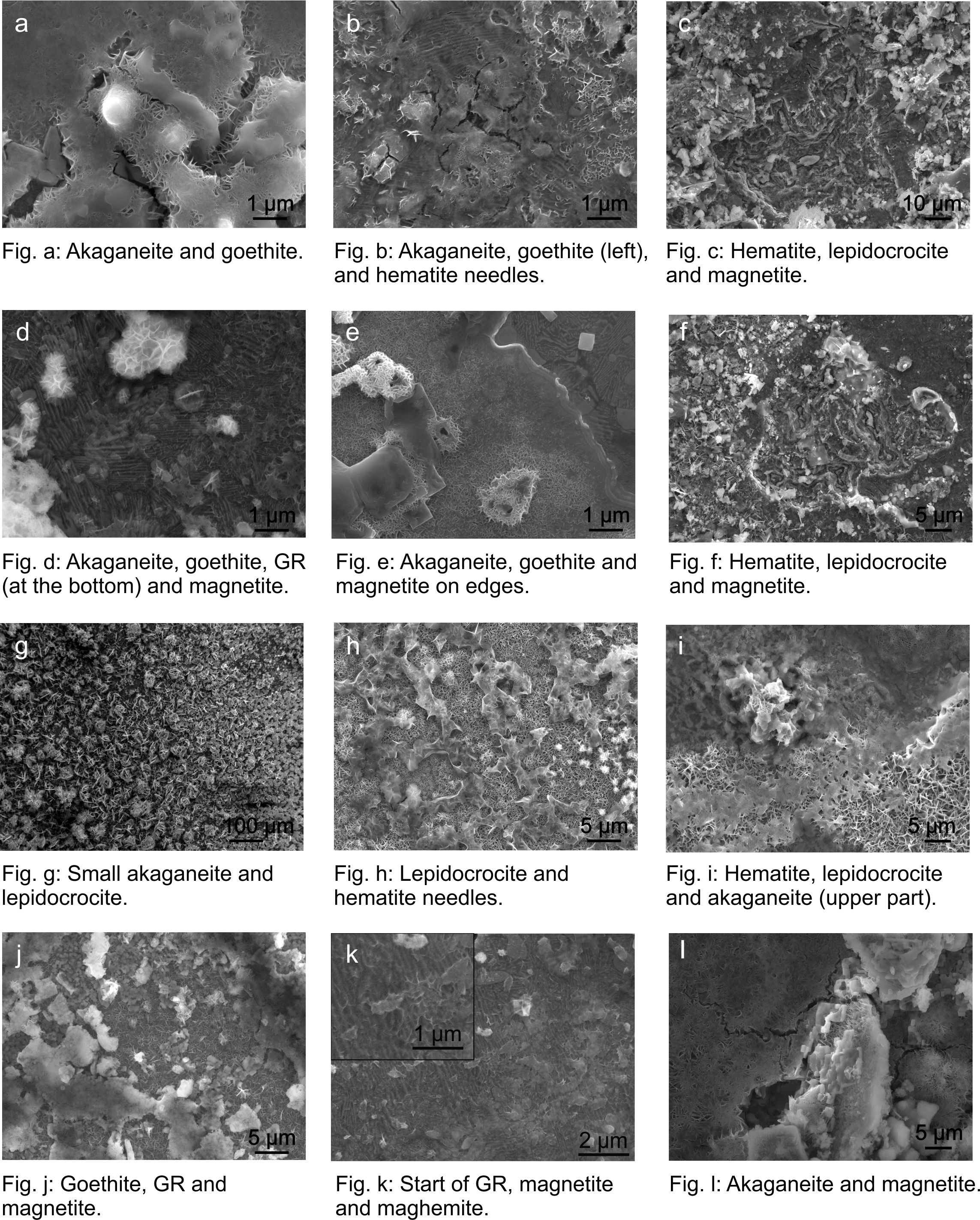

Supplement: Supplementary file 1 [file materials-14-06357-s001.zip › SM/SM4.jpg]

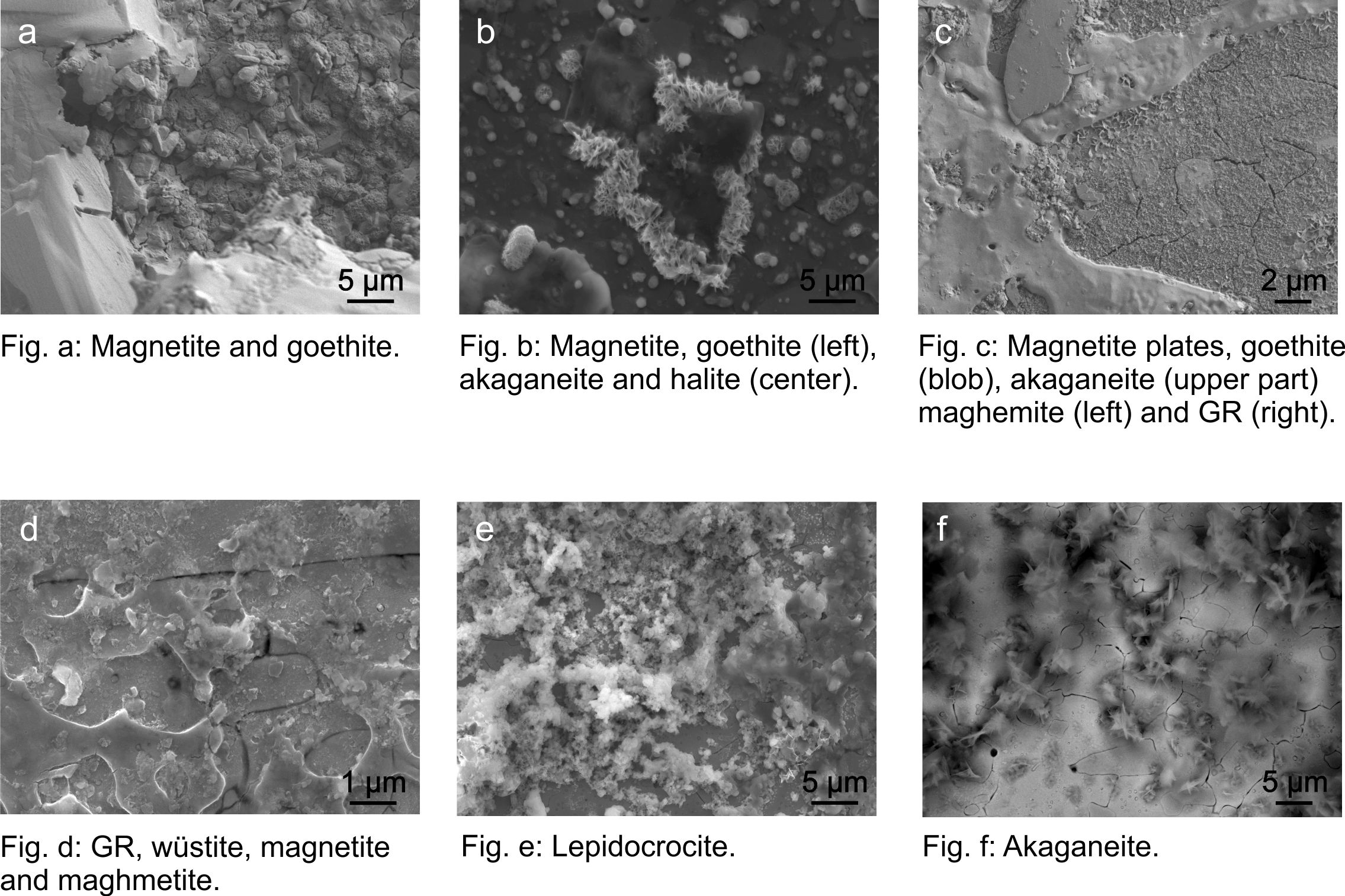

Supplement: Supplementary file 1 [file materials-14-06357-s001.zip › SM/SM5.jpg]
